# Considerations for hormonal therapy in migraine patients: a critical review of current practice

**DOI:** 10.1080/14737175.2023.2296610

**Published:** 2023-12-19

**Authors:** Romy van Lohuizen, Jakob Paungarttner, Christian Lampl, Antoinette MaassenVanDenBrink, Linda Al-Hassany

**Affiliations:** a Division of Vascular Medicine and Pharmacology, Department of Internal Medicine, Erasmus MC University Medical Center, Rotterdam, The Netherlands; b Headache Medical Center Linz, Linz, Austria; c Department of Neurology and Stroke Unit, Koventhospital Barmherzige Brüder Linz, Linz, Austria

**Keywords:** Females, males, menopause, menstruation, migraine, pharmacology, sex hormones, treatment

## Abstract

**Introduction:**

Migraine, a neurovascular headache disorder, is a leading cause of disability worldwide. Within the multifaceted pathophysiology of migraine, hormonal fluctuations play an evident triggering and exacerbating role, pointing toward the need for identification and proper usage of both existing and new hormonal targets in migraine treatment.

**Areas covered:**

With a threefold higher incidence of migraine in women than in men, the authors delve into sex hormone-related events in migraine patients. A comprehensive overview is given of existing hormonal therapies, including oral contraceptives, intrauterine devices, transdermal and subcutaneous estradiol patches, gnRH-agonists, oral testosterone, and 5α reductase inhibitors. The authors discuss their effectiveness and risks, noting their suitability for different patient profiles. Next, novel evolving hormonal treatments, such as oxytocin and prolactin, are explored. Lastly, the authors cover hormonal conditions associated with migraine, such as polycystic ovary syndrome, endometriosis, and transgender persons receiving gender affirming hormone therapy, aiming to provide more personalized and effective solutions for migraine management.

**Expert opinion:**

Rigorous research into both existing and new hormonal targets, as well as the underlying pathophysiology, is needed to support a tailored approach in migraine treatment, in an ongoing effort to alleviate the impact of migraine on individuals and society.

## 1. Introduction

Migraine is a prevalent neurovascular disorder characterized by recurrent moderate-to-severe headache attacks, with an estimated global prevalence of 15% [1,2]. The World Health Organization ranks migraine as the second highest cause of disability in the world, especially in women under the age of 50 [3]. A specific subtype of migraine attacks occurring around menstruation with a close link to hormones is perimenstrual migraine episodes. Perimenstrual migraine affects about 20–25% of females with migraine in the general population, and 22–70% of patients presenting to headache clinics [4]. Perimenstrual migraine is typically characterized by a higher severity and frequency of attacks and more difficulty to treat, compared to hormone-independent migraine episodes [5]. In women diagnosed with menstrual migraine, menstrually related migraine or pure menstrual migraine should be distinguished. About two-thirds of women with migraine experience menstrually related migraine [6,7], where attacks tend to occur around menstruation but are also present at other times of the cycle. However, pure menstrual migraine, in which migraine attacks occur exclusively around menstruation, is a rare condition, affecting less than 1% of women with migraine. Hence, there have been doubts raised regarding the clinical significance of discerning between these subtypes [6,7].

Although the multifaceted pathophysiology of migraine is only partially understood, the initiation of migrainecl attacks is attributed to the activation of the trigeminovascular system. This leads to the release of neuropeptides, in particular calcitonin gene-related peptide (CGRP), causing vasodilation in the meningeal blood vessels and activation of nociceptive transmission [8,9]. For menstrually related migraine, two main triggering mediators of these mechanisms have been identified: estrogen withdrawal and prostaglandin release, which will both be discussed later on.

The hormonal treatments available show unsatisfactory results in many patients, pointing out the requirement of future targets. Moreover, as societal diversification advances (e.g. transgender persons with migraine, patients with comorbidities) increase the complexity of the patient population and the urge for safe and efficacious treatments for these populations is becoming more and more important.

Several overviews have already been published about hormonal treatments for migraine [10–20], mainly focusing on contraceptives and hormone replacement therapies, while missing out on further options like testosterone or GnRH-agonists.

This review aims to present an overview on the relationship between migraine and sex hormones and to provide a summary of current evidence concerning efficacy and safety of all hormonal treatments available with a focus on clinical studies. Another focus will be on migraine treatment in certain subpopulations with a special hormonal landscape like transgender persons or women suffering from endometriosis or polycystic ovary syndrome. While this review will also provide a glimpse into future therapeutic options for migraine, it is of particular importance to emphasize the relevance of further research and rigorous studies on this topic. Because current therapy options often show limited effectiveness, an extended knowledge about the pathophysiology and pharmacological aspects of this disorder will be crucial to improve the quality of life of those affected.

## 2. Literature search

A search strategy was developed together with a librarian of the Erasmus MC Medical Library. The search strategy included the terms ‘migraine,’ ‘(peri)menopause,’ ‘hormone/hormonal treatment’ (exact search strings are included in the Appendix) and focused on clinical studies. The following databases were searched on the 22^nd^ of June 2023 without time restriction: Medline ALL, Embase, Web of Science Core Collection, Cochrane Central Register of Controlled Trials, and Google Scholar (100 top-ranked). While no systematic selection of articles was performed, we additionally included references of retrieved articles.

## 3. Sex hormone related events in migraine patients

**Figure 1.**
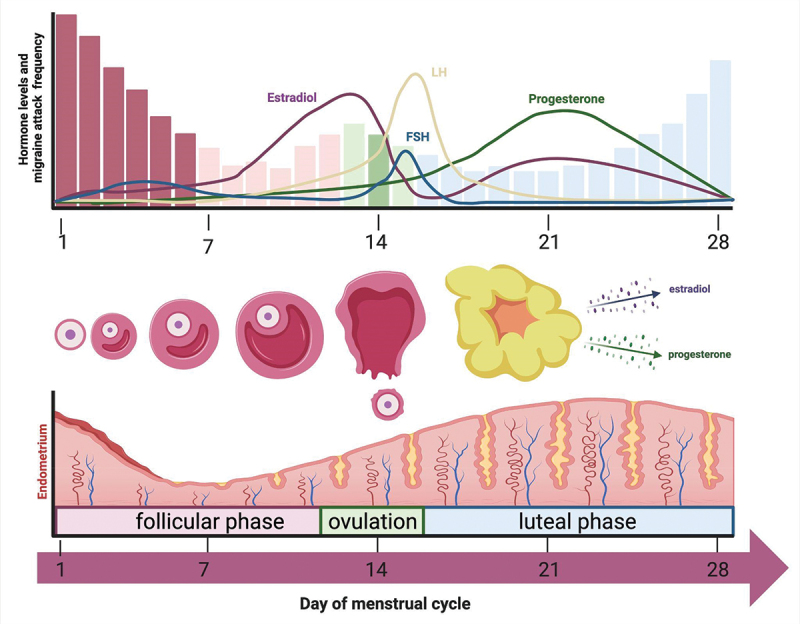
Menstrual cycle and migraine frequency. In this figure, serum levels of hormones and their effect on the endometrium over the course of the menstrual cycle are depicted, as well as the concurrence of fluctuations in the incidence of migraine attacks in women with menstrual migraine. Lasting around 28 days, the cycle starts with the release of FSH, stimulating the growth of ovarian follicles. The maturation of these follicles leads to an increasing production of estradiol, which triggers a surge in LH and causes ovulation. After ovulation, the ruptured follicle transforms into the corpus luteum which produces progesterone and prepares the endometrium for possible fertilization. If fertilization does not occur, the corpus luteum breaks down progesterone and estradiol levels drop, which triggers menstruation and the start of a new menstrual cycle [23]. The decline of estradiol, also referred to as estradiol-withdrawal, is also thought to precipitate a migraine attack without aura in women with menstrual migraine [24]. The figure is based on the data from Martin et al. [25] and MacGregor et al. [26] and was created using BioRender.

Sex hormones, including estrogen, progesterone, and testosterone, play a multifaceted role in migraine pathophysiology [21]. MacGregor et al. showed that in the 5 days preceding menstruation, the likelihood of suffering a migraine attack was increased by 25% [22]. This climbed up to 71% in the 2 days preceding menstruation. On the first day of menstruation and 5 days after the risk was increased by about 100%. Figure 1 depicts serum levels of sex hormones and their effect on the endometrium over the course of the menstrual cycle, as well as their concurrence with fluctuations in incidence of migraine attacks in women with menstrual migraine.

The key role of sex hormones in migraine can also be comprehended by analyzing how different life events influence migraine frequency. Before puberty, differences between the activity of sex hormones are less pronounced, and there is equal prevalence of migraine in both sexes [27]. After menarche, there is a sharp increase in migraine prevalence in women, leading to a shift in male-to-female ratio [28]. When it comes to pregnancy, about 50–75% of women with migraine experience a reduction in migraine attacks and pain intensity, especially in the second and third trimesters. While the headache frequency can increase in the postpartum period, breastfeeding seems to have a protective effect [29]. When leaving the fertile years and moving on to menopause, the perimenopausal period is associated with another increase in frequency, pain severity, and treatment response of migraine while experiencing a decline in those factors after natural menopause [30].

The entirety of mechanisms involving different hormones have not been fully elucidated. However, several studies have shown that fluctuating levels of different sex hormones can either trigger a migraine attack or have a protective influence [31]. The evidence concerning the role of several hormones will be explained shortly.

### 3.1. Estrogen

A key theory involving hormonal influences in migraine is the ‘estrogen withdrawal hypothesis,’ stating that changes in serum estradiol levels, especially drops in estradiol during the luteal phase just before menstruation, triggers menstrual migraines in susceptible women [24,26,31]. This increase in the occurrence of migraine attacks is most likely to be linked to an interaction between sex hormone fluctuations and CGRP, as demonstrated by animal and human preclinical models [32]. Fluctuations of mainly estrogen modulate CGRP release in the peripheral and central trigeminovascular system, as reviewed by Labastida-Ramérez et al. [32].

Valdemaarson et al. studied plasma levels of CGRP in a healthy population and found significantly higher levels in females than in males, while women using contraceptives had significantly higher levels than women without [33]. The correlation between estrogen levels and CGRP levels during menstruation was also demonstrated by Raffaelli et al. They measured CGRP levels in plasma and tear fluid during menstruation (i.e. when serum estrogen levels are low) and showed that women suffering from migraine had elevated CGRP levels compared to healthy controls [34].

An observational study found that the rate of decline of conjugated urinary estrogens in migraine patients in the late luteal phase is faster compared to controls, while there was no difference in the rate of estrogen withdrawal after the periovulatory peak. Additionally, the authors demonstrated that within migraine patients, the rate of conjugated urinary estrogens decline remained consistent, regardless of whether a headache occurred during the menstrual cycle. Researchers then proposed a ‘two-hit’ hypothesis, indicating that the faster estrogen decline could be an endogenous feature of women with migraine, leading to neuroendocrine vulnerability which may facilitate migraine attacks by common triggers, like alcohol or stress [35].

Ibrahimi et al. performed a case–control study to examine the dermal blood flow responses to capsaicin and electrical stimulation on days 1–2 and 19–21 of the cycle in women suffering from menstrual migraine. They found that in the control group, responses to capsaicin were higher on days 1–2 than on days 19–21, a finding which was not detected in the migraine group (where responses were similar over the cycle), leading to the hypothesis of reduced trigeminovascular cyclicity in women suffering from menstrual migraine. Interestingly, they also found that estradiol serum levels on days 19–21 were higher in healthy controls (75 ± 8 pg/mL) than in women with menstrually related migraine (52 ± 4 pg/mL) [36].

Serum estradiol does not only play a key role in migraine in females but may also be involved in pathophysiology of male migraineurs. Van Oosterhout et al. measured the 17β-estradiol and calculated free testosterone in serum of male migraineurs and healthy controls. They found that the migraine group had significantly higher interictal 17β-estradiol (96.8 ± 6.1 vs 69.1 ± 5.6 pmol/L) levels and a lower 17β-estradiol/free testosterone ratio (3.9 ± 0.4 vs 5.0 ± 0.3) compared to controls [37].

Pringsheim et al. [38] examined the effect of antiandrogens and estrogens used by male to female transsexuals. They included 50 transsexuals, each of whom had to fill out a questionnaire consisting of 8 questions regarding the characteristics of their headaches. The results showed that the prevalence of migraine in the male to female transsexuals was similar to the prevalence expected for genetic females. Moreover, 54% of those suffering from migraine reported visual aura, a phenomenon which could disappear after reducing the dosage of estrogen [39].

### 3.2. Progesterone

Progesterone is believed to have a protective effect against migraine attacks by modulating nociception and downregulating estrogen receptors [40,41]. Progesterone, which is mainly active during the luteal phase [42], is believed to hold a protective role in migraine. Similar to estrogen, progesterone plasma levels decrease before menstruation [43]. However, two studies [44,45] assessed the role of withdrawal of progesterone and estrogen, clearly showing the worsening effect of estradiol withdrawal, while the withdrawal of progesterone did not have any worsening effect on the course of migraine.

While the worsening effect could not be demonstrated, a possible pathway of progesterone being protective against menstrual migraine attacks was found. This might be due to reducing activation in the trigeminal nucleus caudalis and causing an antinociceptive effect [40]. Additionally, the neurosteroid allopregnanolone, which is an active metabolite of progesterone and acts as a positive modulator of Gamma-Aminobutyric Acid (GABA) receptors, has shown antinociceptive effects in animal studies [43,46,47]. This brought authors to consider the hypothesis that progesterone and its metabolite, allopregnanolone, hold the potential to be beneficial in migraine by modulating pain perception. However, the administration of progesterone does not prevent migraine attacks [44], leading to the opinion of progesterone not being a major factor in triggering these attacks [43].

### 3.3. Testosterone

The role of the neuroactive steroid testosterone in the modulation of migraine headaches is less well understood compared to estrogen and progesterone, although it may play a role in cerebral blood flow and serotonergic tone [48]. Testosterone has been shown to have an endothelium-independent vasodilatory role in preclinical studies as reviewed by Herring et al. [49].

A recent study found that the prenatal estrogen–testosterone balance might be a risk factor for migraine in adults [50]. Females suffering from migraine were allegedly exposed to higher testosterone levels relative to estrogen in the prenatal period, while males suffering from migraine were presumably exposed to higher estrogen levels relative to testosterone, during their prenatal period.

In mice, it has been shown that the administration of testosterone plays an important role in the suppression of cortical spreading depression in mice with familial hemiplegic migraine [51]. In addition, in healthy men, testosterone – but not estradiol – has been demonstrated to negatively correlate with serotonin 4 receptor levels [52].

Further, a small study consisting of 15 postmenopausal women not taking estrogens found no significant differences in serum levels of androstenedione, total testosterone, and free testosterone between those with migraine and healthy controls [53]. In addition, Shields et al. measured the testosterone levels in men with chronic migraine. They found that men suffering from chronic migraine had significantly lower mean testosterone levels than age-matched controls (322 ng/dL vs. 384 ng/dL) [54].

### 3.4. Gonadotropin releasing hormone

There is no evidence regarding the role of gonadotropin releasing hormone (GnRH) in the course of migraine. However, the suppression of gonadotropins (luteinizing hormone (LH) and follicle-stimulating hormone (FSH)) due to the administration of large doses of GnRH appears to have beneficial effects on migraine severity, as discussed in the treatment section [55].

### 3.5. The role of the hypothalamic-pituitary-ovarian axis

The hypothalamic-pituitary-ovarian axis describes a tight endocrine system that plays a central role in regulating female reproductive function [56]. The pulsatile release of GnRH leads to the secretion of FSH and LH from the pituitary gland which subsequently causes the release of estrogen and progesterone from the ovaries. Over the course of the menstrual cycle, the pulsatile secretion of GnRH results in different serum levels of the hormones involved, including LH and FSH [57].

The above mentioned system and its role in migraine were examined by Facchinetti et al., who measured LH, FSH, prolactin, estradiol, and progesterone before and after the prophylactic treatment of dihydroergotamine in women with perimenstrual migraine. The plasma levels of LH, FSH, and prolactin were similar between women with migraine and controls and were unaffected by the prophylactic treatment with dihydroergotamine. However, women with migraine showed reduced progesterone levels over the entire course of the luteal phase, which was accompanied by higher estradiol levels, which lead to a progesterone/estradiol ratio which significantly disturbed ovarian steroid secretion. Based on these data, it can be inferred that there is an impairment of ovarian secretion in the context of perimenstrual migraine [58].

## 4. Hormonal treatment and management

**Figure 2.**
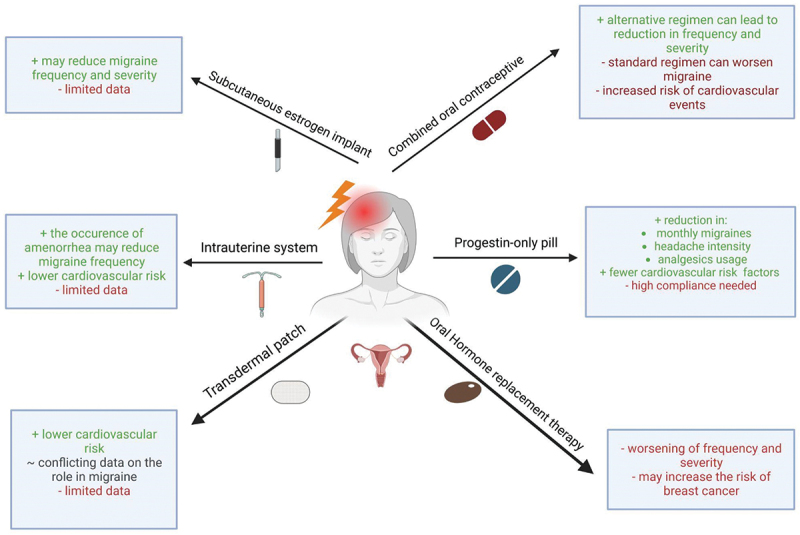
Hormonal treatment of migraine, their advantages and disadvantages. this figure provides an overview of existing hormonal therapies, their benefits, risks and potential challenges. Figure was created using BioRender.

Hormonal treatment for migraine represents a diverse array of approaches aimed at modulating hormonal levels and their effects on frequency, severity, and duration of migraine attacks. This section highlights the existing hormonal therapies in migraine, their mechanisms of action, efficacy, side effects, challenges, and considerations for patient selection. Table 1 shows a summary of results of the important mentioned studies in this chapter. Figure 2 additionally provides a compact overview of the existing hormonal therapies, their benefits, risks, and potential challenges. It is important to note that the treatment options presented in this chapter have mostly been developed for contraception and other hormonal treatments. Considering the fact that migraine belongs to the most disabling diseases in the world, especially in the subgroup of younger women, it might seem unimaginable that there are no specific and targeted treatment options and clinicians are forced to use medication developed for totally different indications. A condition that on the one hand prompts contemplation but on the other hand underlines the importance of further research on more effective and safe treatment options.

**Table 1.** Summary of the main original studies investigating the effect of hormonal treatments. Papers focusing on adverse events or case series are not included in this summary.

Study	Study type/design	Number of included migraine patients	Treatment	Migraine phenotype	Treatment duration	Outcome(s)/main findings
Merki-Feld et al. [59]	Prospective controlled trial	150, comprising of: 98 in the intervention group and 36 in the control group	75 µg desogestrel daily (progestin-only pill)	Menstrual migraine	180 days	↓ Monthly migraine days ↓ Headache intensity ↓ Monthly days using pain medication ↓ Monthly number of triptans
Aegidius et al. [60]	Cross-sectional population-based study	2,923	Combined oral contraceptives	Not specified	n.a.	↑ Headache, especially migraine, prevalence
Warhurst et al. [61]	Systematic review and meta-analysis	165 (4 studies)	Progestin-only pill	Migraine with aura, migraine without aura and menstrual migraine	180 days	↓ Monthly migraine attacks ↓ Monthly migraine days ↓ Duration of migraine ↓ Headache intensity ↓ Monthly days using pain medication ↓ Number of triptans used over 3 months
Lieba-Samal et al. [62]	Prospective diary study	184	Combined oral contraceptives	Perimenstrual migraine	90 days	↑ Migraine prevalence
De Leo et al. [63]	Prospective randomized control study	60	Combined orale contraception: ethinyl E [2] 20 μg/drospirenone 3 mg Comparison of a 21/7 regimen with a 24/4 regimen (days of contraceptive pill/hormone free interval)	Pure menstrual migraine without aura	3 months	*24/4 regimen*: ↓ Headache intensity ↓ Duration of migraine
Nappi et al. [64]	Prospective diary based study	32	Estradiol and dienogest (a progestin) which was administered for 6 cycles. The pill dosages differed during the cycle, only the pills to be taken on day 27 and 28 were hormone free.	Menstrually related migraine	6 menstrual cycles	↓ Migraine days ↓ Duration of migraine ↓ Number of analgesics
Sulak et al. [65]	Prospective cohort study	111	Combined oral contraceptives for 21 days + 7 day period of placebo, followed by a combination of 30 μg ethinylestradiol and 3 mg drospirenone continuously for 168 days, without any hormone-free interval	Menstrually related migraine	28 + 168 days	↓ Number of days with reduced productivity ↓ Number of days unable to do housework ↓ Duration of migraine ↓ Number of migraines persisting despite medication
Coffee et al. [66]	Prospective pilot study	30	Combined contraceptive containing ethinylestradiol and levonorgestrel	Menstrually related migraine	Baseline period of two menstrual cycles, followed by 168 days on the combined oral contraceptive	↓ Daily headache scores
Calhoun [67]	Pilot open-label study (in which a 28-day menstrual cycle diary was kept)	11	Estrogen supplementation during otherwise hormone free interval: 20 µg ethinylestradiol on day 1 to 21 and 0.9 mg conjugated equine estrogens on day 22 to 28	Menstrually related migraine	28 days	↓ Number of headache days ↓ Weighted headache score
Nappi et al. [68]	Randomized prospective study	40	Tibolone (*n* = 20) *versus* conventional hormone replacement therapy (*n* = 20)	Migraine without aura (and episodic tension-type headache)	3 months	*Tibolone*: → Migraine days without aura ↓ Number of hours during which pain intensity prohibited daily activities ↓ Monthly number of analgesics *Hormone replacement therapy*: ↑ Monthly migraine days.
Misakian et al. [69]	Cross-sectional study (part of the Women’s Health study)	Migraine group: 1,909 No migraine group: 15,198	Hormone replacement therapy	Migraine (not specified)	n.a.	↑ Migraine prevalence
Kim et al. [70]	Population-based study	Migraine group: 150,802 Control group: 963,940	Hormone replacement therapy (HRT)	Spontaneous postmenopausal women	Analyses were based on HRT duration (none, <2 years, <5 years, >5 years, and unknown)	↑ Risk of migraine in postmenopausal women using HRT
Vetvik et al. [71]	Interview study	49	Any type of hormonal contraceptives	Menstrual migraine without aura	N.a.	The appearance of amenorrhea during the use of contraceptives: ↓ frequency of migraine without aura (compared to women with menstrual/withdrawal bleeds)
Almén-Christensson et al. [72]	Randomized, double-blinded, placebo-controlled crossover study	27	Transdermal estradiol patch/gel containing 100 μg 17β-estradiol	Menstrual migraine	Medical patch for 3 menstrual cycles, before or after placebo patch for 3 menstrual cycles	→ Number of migraine attacks → Severity of migraine attacks → Duration of migraine attacks
Guidotti et al. (155)	Pilot, open-label, non-randomized, parallel group study	38	2.5 mg oral frovatriptan (*n* = 14), 500 mg oral naproxen sodium (*n* = 14), 25 μg transdermal estradiol (*n* = 10)	History of menstrual migraine	A control period without prophylaxis and a treatment period (number of days not specified)	Frovatriptan use is superior to naproxen and estrogen (which shows no significant effect)
Dennerstein et al. [73]	Double-blind crossover study	22 (18 completed the study)	Transdermal estradiol for two consecutive cycles followed by two placebo cycles, or the other way around	Menstrual migraine with or without aura	7 cycles (two assessment, four treatment and one follow up cycle	↓ Headache frequency during the estradiol cycles *versus* placebo cycle ↓ Analgesic use during the estradiol cycle *versus* placebo cycle
De Lignières et al. [74]	Double-blind, placebo controlled crossover study	18	9 women were administered the drugs in the order estradiol gel, placebo, estradiol gel and 9 women in the order placebo, estradiol gel, placebo (change in treatment per cycle)	Menstrual migraine	Three menstrual cycles	*During the estradiol cycle*: ↓ menstrual migraine attacks duration of attacks ↓ severity of attacks ↓ aspirin use as an acute treatment
MacGregor et al. [75]	Double-blind placebo-controlled crossover study	35	Placebo gel or estradiol gel for the first 3 cycles, after that a switch of these for another 3 cycles	Menstrual migraine or menstrually related migraine (numbers per group not specified)	6 menstrual cycles	*During the estradiol cycles*: ↓ Migraine days ↓ Migraine severity ↓ Nausea *After stopping the estradiol treatment*: - First 5 days: ↑ migraine frequency - After five days: normal state
Calhoun et al. [76]	Retrospective database review	23	Vaginal ring containing 0.120 mg etonogestrel/15 μg ethinyl estradiol	Migraine with aura and menstrually related migraine, with reported high headache burden	Extended regimen of a vaginal ring	*Use of extended-cycle dosing*: ↓ Aura times per month ↓ Menstrually related migraine (disappeared in 91.3%)
Magos et al. [77]	Prospective study, not placebo controlled	24	Subcutaneous estradiol implants, at a high enough dose to inhibit ovulation	Menstrual migraine	Five years	↓ Number of migraine attackss ↓ Severity of migraine attacks ↑ Improvement of symptoms (11 became completely headache free)
Murray et al. [55]	Prospective study, not blinded or placebo-controlled	5	Leuprolide acetate – a GnRH-agonist − 3.75 mg i.m. monthly for 10 months. After 5 months into the period, researchers started to administer ‘add-back’ therapy, consisting of transdermal ethinylestradiol (0.1 mg) and oral medroxyprogesterone acetate (2.5 mg), both daily, to avoid menopausal symptoms and bone loss.	Severe menstrual migraine	Two month observation, followed by 10 month treatment	↓ Mean headache score, during both first five months and later during the ‘add-back’ therapy
Glaser et al. [48]	Prospective pilot study without control group	27	Testosterone pellets, subcutaneously implanted	Migraine and symptoms of androgen deficiency, of which 16 pre- and 11 postmenopausal	Three months of therapy	↓ Headache severity

*Treatment outcomes are depicted as follows*: ↓= a decrease; ↑ = an increase; → = no difference.

### 4.1. Cardiovascular risk in migraine patients using hormonal treatments

There are numerous options for hormonal treatment in migraine patients. However, these options drastically differ in effectiveness and risk profile. Therefore, the following aspects have to be taken into account, when considering hormonal treatment in migraine:

It is widely known that migraine, especially with aura, is associated with an increased risk of stroke and other major cardiovascular events [78,79]. A recently published meta-analysis including 40 studies (18 case–control studies and 22 cohort studies) and 6,279,823 participants indicates that subjects suffering from migraine with aura are particularly affected (RR 2.17 95% CI 1.78–2.64). However, subjects suffering from migraine without aura have a higher risk as well (RR 1.34; 95% CI 1.11–1.62) [80]. These numbers were confirmed by another meta-analysis [78], showing an elevated risk for stroke (HR 1.30, 95% CI 1.07–1.60), myocardial infarction (HR 1.36, 95% CI 1.23–1.51), and even cardiovascular mortality (HR 1.27, 95% CI 1.14–1.42) among migraine patients.

Migraine with aura is also associated with other risk factors for ischemic stroke such as patent foramen ovale, lupus erythematosus, and antiphospholipid syndrome [11,79].

The elevated risk of stroke in people suffering from migraine, is even further increased by the use of certain hormonal treatments, especially estrogen-containing prescriptions which lead to an increase in some components of the procoagulant pathway [11] Champaloux et al. [81] performed a nested case–control study examining associations between migraine, combined hormonal contraceptives and stroke in females aged 15–49. They included 25.887 ischemic strokes and found that the risk of ischemic stroke was highest among women suffering from migraine with aura and using combined hormonal contraceptives (OR = 6.1; 3.1–12.1; 95% CI). The risk for women suffering from migraine without aura and using combined hormonal contraceptives was less elevated (OR = 1.8; 1.1–2.9; 95% CI). Furthermore, the risk is even further increased in young women who suffer from migraine with aura, who smoke and use oral contraceptives [82].

Therefore, given that migraine with aura and combined hormonal contraceptives are both independent risk factors for cardiovascular diseases, especially ischemic stroke, precaution by clinicians is warranted. The following chapter aims to give an overview of the hormonal treatments available, describing the effectiveness and associated risks for migraine treatment as presented in Figure 2.

### 4.2. Progestin-only pill

The progestin‐only pill, also known as ‘mini-pill,’ contains low doses of a progestin, mostly levonorgestrel, norethindrone, ethynodiol diacetate, or desogestrel. The dose of progestin is lower than in combined oral contraceptives [83]. Several studies report effectiveness of the progestin-only pill in menstrual migraine. It is used continuously, which leads to the suppression of ovulation, smoothing hormonal fluctuations [59].

A recent meta-analysis included four studies which examined the effect of 75 µg desogestrel in premenopausal women with migraine. Two studies additionally compared the progestin-only pill to a combined oral contraceptive, containing 150 µg desogestrel and 20 µg ethinylestradiol, after 90 and 180 days treatment. A mean reduction of migraine attacks per month was found by − 0.96 [−1.41, −0.52], of monthly migraine days by − 2.05 [−2.92, −1.18], a reduction in mean duration by −4.28 hours [−6.56, −2.00] and a reduced triptan use per 90 days by − 2.49 [−4.70, −0.28] [61].

Merki-Feld et al. performed a prospective controlled trial in which they administered 75 µg desogestrel daily to female migraine patients (*n* = 98), who opted for this form of contraception themselves. The control group comprised women who continued their usual contraceptive (*n* = 36). They showed that after 90 and 180 days, there was a significant decrease in migraine days (7.3 vs. 5.0 vs. 5.4), headache intensity (18.3 vs. 16.0 vs. 13.6), days using pain medication (7.8 vs. 5.8 vs. 5.6), and monthly number of triptans used (14.7 vs. 10.2 vs. 10.4) [59].

The use of the progestin-only pill over combined hormonal contraceptives found no significant differences in the risk of adverse events, according to a systematic review and meta-analysis by Warhurst et al. The only difference found was an elevated risk of prolonged bleeding in the progestin-only pill group [61].

In a consensus statement by the European Headache Federation (EHF) and the European Society of Contraception and Reproductive Health the use of progestin-only pill was suggested for women with migraine with aura or women with migraine without aura and additional cardiovascular risk factors [12,84].

### 4.3. Combined oral contraception

Combined oral contraceptives have been used for contraception over decades, with a high variability of doses, hormones, and regimes. The main component is usually ethinylestradiol, combined with a progestogenic component of different dosages. The standard regimen is 21/7 meaning to take the contraceptive pill for 21 consecutive days, followed by a 7-day break, during which menstruation typically occurs and which provides a hormone free interval of 7 days [85]. Several studies have shown an association between combined oral contraceptives and an increase of migraine frequency, especially in the hormone free interval [60,62,86].

However, a change of the regimen in order to reduce or even skip the hormone free interval has shown positive effects. A prospective randomized study consisting of 60 women suffering from pure menstrual migraine without aura compared the standard regimen of 21/7 (Group A) with a 24/4 (Group B) regimen, i.e. 24 days of contraceptive pill followed by a four-day hormone free interval. Group B showed greater reduction concerning the intensity and duration of menstrual migraine. These improvements were shown to be present from the first combined oral contraceptive cycle and increased gradually throughout the study period [63].

Shortening the pill-free interval was also examined by Nappi et al. [64]. In their prospective diary-based study, 32 women suffering from menstrually related migraine were included. After an observational period of three menstrual cycles, patients were put on a contraceptive containing estradiol and dienogest (a progestin) which was administered for six cycles. The pill dosages differed during the cycle, only the pills to be taken on days 27 and 28 were hormone free. Follow-up evaluations were performed after three and six cycles. The number of migraine days declined from 2.7 ± 0.9 days at baseline to 2.2 ± 0.7 after the third and 2.0 ± 0.7 after the sixth cycle. Moreover, duration of headaches dropped around 44.7% at cycle 3 and 46.1% at cycle 6, compared to baseline. The number of analgesics taken decreased from 4.7 ± 1.1 to 3.3 ± 0.7 at cycle 3 and 2.9 ± 0.6 at cycle 6 [64].

Sulak et al. performed a prospective cohort study in which 111 subjects with menstrually related migraine were administered combined oral contraceptives for 21 days followed by a 7-day period with placebo. Afterward, the subjects received a combination of 30 μg ethinylestradiol and 3 mg drospirenone continuously for 168 days, without any hormone-free interval. The data was collected by the use of headache questionnaires. This questionnaire consisted of eight questions, of which six improved significantly during the 168 days, compared to the standard 21/7 cycle. The number of days with reduced productivity significantly decreased by 48.5%, the number of days unable to do housework by 50%. There was also a significant reduction concerning the average duration of headache by 39.5% and number of headaches that persisted despite medication by 39.5% [65].

The extended regimen in combined oral contraceptives was also examined by Coffee et al. in a prospective pilot study. After a baseline period of two menstrual cycles, they placed 30 women suffering from menstrually related migraine on an oral contraceptive, containing ethinylestradiol and levonorgestrel for 168 days. Women experiencing breakthrough bleeding after initiation of the extended regimen were put on a 4-day hormone-free interval to resolve the bleeding. Each participant was advised to take a hormone-free interval after 84 days and to report the presence and characteristics of their headaches using a headache diary and measured by migraine disability assessment score (MIDAS). After 168 days, they found a decrease in daily headache scores from 1.29 ± 0.1 to 1.10 ± 0.14 [66].

Another pilot study examined the effect of estrogen supplementation during the otherwise hormone-free interval. Eleven patients received 20 µg ethinylestradiol on day 1 to 21 and 0.9 mg conjugated equine estrogens on day 22 to 28. This resulted in a mean reduction of headache days by 76.3%, and an average reduction of headache severity by 77.9% [67].

Combined oral contraceptives increase the risk of venous thrombosis, which could result in pulmonary embolism or stroke. A network meta-analysis of Stegemann et al. included 26 studies, in which different combined oral contraceptives with different dosages were examined. They found that every type of combined oral contraceptives increases the risk of venous thrombosis; however, they showed that combinations using levonorgestrel had a 50–80% lower risk and that the dosage of ethinylestradiol is directly proportional to the risk of venous thrombosis [87].

Despite above mentioned evidence, large trials supporting the efficacy of the oral contraceptive pill as prophylactic migraine therapy are missing. Hence, a large clinical trial was started, the ‘Migraine WHAT-study,’ which stands for “Women, Hormones, Attacks, and Treatment (ClinicalTrials.gov ID: NCT04007874), and is still ongoing. The study lets women with regular menstrual cycles document their headache symptoms and menstrual-related symptoms for 3 months, using e-diaries. Furthermore, sex hormones are measured in blood, urine, and saliva at various points in the menstrual cycle in women who experience migraine attacks during menstruation or perimenopause. This is followed by an open-label randomized controlled trial to study the efficacy of continuous daily use of ethinylestradiol/levonorgestrel (30/150 µg/day) compared with vitamin E (400 IU/day) in the treatment of women with menstrually related and perimenopausal migraine [88,89].

The risk of stroke for women aged 20–44 years who suffer from migraine without aura or migraine with aura and are using hormonal contraception was addressed in a consensus statement from the EHF and European Society of Contraception and Reproductive Health. They performed a systematic literature search which included 63 studies in their final analysis. They concluded that the baseline risk of women suffering a stroke is 2.5/100,000, while the risk for women using combined hormonal contraception is 6.3/100,000. This risk certainly increases in women with migraine with aura, who have a risk of 5.9/100,000 without hormonal contraception and a risk of 36.9/100,000 using hormonal contraception. When suffering from migraine without aura, the risk is 4.0/100,000 without, and 25.4/100.000 with hormonal contraception. They also stated that the risk increases with a higher dosage of ethinylestradiol [84].

### 4.4. Oral hormone replacement therapy

Hormone replacement therapy (HRT), prescribed in order to alleviate the vasomotor symptoms during the menopausal transition, usually consists of a combination of estrogens and gestagens. Estrogen monotherapies are contraindicated because of the increased risk of endometrial cancer. This risk can be decreased by adding a gestagenic component [90].

Several studies have shown that HRT can worsen migraine in postmenopausal women. Nappi et al. compared the effect of tibolone with the conventional HRT in 40 headache patients. They found that tibolone did not reduce the total number of migraine days without aura but did significantly reduce the number of hours during which pain intensity prohibited daily activities and the number of analgesics (−26.1%) taken after 3 months. However, they also showed that the subjects receiving the conventional HRT had a significant increase in migraine days [68].

In a cross-sectional study, which was conducted as part of the Women’s Health study, researchers found that current users of HRT were about 40% more likely to suffer from migraine than people who had never used HRT. After correction of confounding variables, this association was still significant in nearly all HRT regimens [69].

Kim et al. [70] used data from a South-Korean population-based study to examine the influence of endogenous and exogenous hormonal factors on migraine in 1.114.742 spontaneous postmenopausal women without a history of migraine. All included women were divided into a migraine group and a control group, depending on migraine diagnosis assessed during the follow-up period. They found a significantly higher risk of migraine in postmenopausal women using HRT than in individuals not using HRT. The groups using HRT <2 years had an HR of 1.194 (95% CI 1.176–1.213), while individuals using HRT <5 years had an HR of 1.165 (95% CI 1.137–1.193) and those using HRT ≥5 years had 1.194 (95% CI 1.162–1.227) [70].

While evidence regarding the effects of HRT on aura symptoms is scarce, a case series of four women receiving HRT showed that higher doses of estrogen can trigger migraine aura. After reducing the estrogen doses, the aura symptoms disappeared [39].

Tibolone, an agonist of estrogen, progesterone, and androgen receptors, has also shown promising effects in primary headaches in postmenopausal women [68,91].

The association between HRT and breast cancer has been a highly researched topic for many years. There are data available showing an increased risk of breast cancer while not having any cardiovascular benefits [92]. This led to an intensification of research concerning the relationship between HRT and breast cancer, resulting in the publication of controversial data [93,94].

Vinogradova et al. [93] recently performed two nested case–control studies including 98.611 women with breast cancer and 457.498 controls in total. They could show that long-term use of ≥5 years of estrogen or combined estrogen/progestogen therapy increased the risk of breast cancer compared to never users (adjusted odds ratio 1.15 (95% CI 1.09–1.21) and 1.79 (95% CI 1.73–1.85). It is important to mention that the risk of breast cancer compared to never users of HRT varied significantly between the different groups of progestagens.

In a prospective cohort study [91] which included 80.377 women and compared the risk of breast cancer in those using HRT to subjects who never used HRT, a beneficial risk profile for formulas using estrogen-progesterone (RR 1.00 (0.83–1.22), 95% CI) and estrogen-dydrogesterone (RR 1.16 (0.94–1.43), 95% CI) compared to formulas using other progestagens (RR 1.69 (1.50–1.91), 95% CI) were found.

Further, Anderson et al. [95] performed a randomized, double-blinded, placebo-controlled trial in which they found that estrogen monotherapies in hysterectomized women significantly decreased the risk of breast cancer (HR, 0.77 95% CI 0.62–0.95).

### 4.5. Levonorgestrel intrauterine system

The levonorgestrel intrauterine system appears to be a good alternative to the standard hormone therapy. It can be either used as a safe way of contraception in fertile women, but also as a part of HRT, providing endometrial protection, while the dose of oral estrogen can be adjusted according to the vasomotor symptoms in perimenopausal women [96]. However, there is only one study addressing the effect of levonorgestrel intrauterine system on migraine [71]. They found that the appearance of amenorrhea during the use of contraceptives led to a reduction of the frequency of migraine without aura compared to women with menstrual/withdrawal bleeds (OR 3.5, 95% CI 1.1–11.4). Women in the amenorrhea group mainly used the levonorgestrel intrauterine system (19/23). A possible explanation for this is the fact that amenorrhea normally appears 6–12 months after insertion, reducing the level of prostaglandins reaching the systemic circulation and therefore reducing migraine frequency.

The levonorgestrel intrauterine system is also considered a ‘no-risk product’ concerning the risk of stroke, by the European Headache Federation (EHF) and European Society of Contraception and Reproductive Health (ESC) consensus statement [84].

As there are only limited data on methods, future studies should address the underlying pathophysiological role of amenorrhea in migraine [71].

### 4.6. Transdermal estradiol patch/gel

Transdermal estradiol is usually prescribed for HRT in postmenopausal women, showing beneficial effects on the vaginal epithelium, bone resorption, and vasomotor symptoms. Moreover, transdermal administration is easy and well tolerated [12]. However, studies performed on the effect of transdermal estradiol in migraine have shown conflicting results. Almén-Christensson et al. [72] performed a randomized, double-blinded, placebo-controlled crossover study, using 100 μg 17β-estradiol in 27 fertile women with menstrual migraine. Both groups received the patch over a period of three menstrual cycles, before or after receiving placebo patches for three cycles. They found no difference between the estradiol and placebo patches on the duration, number or severity of menstrual migraine attacks, although both groups reported an improvement in the number of migraine attacks.

Dennerstein et al. performed a double-blind crossover study, consisting of seven cycles (two assessment, four treatment and one follow-up cycle). During the treatment cycles, the participants, who suffered from menstrual migraine with or without aura, either received transdermal estradiol for two consecutive cycles followed by two placebo cycles or the other way around. Results showed a significant reduction in headache frequency during the estradiol cycles compared to placebo. The analgesic use was also reported to be lower during the estradiol cycle than during the placebo cycle [73].

A double-blind, placebo-controlled crossover study including 18 women with menstrual migraine and regular menstrual cycles investigated the effect of transdermal estradiol gel on menstrual migraine [74]. The study consisted of three consecutive menstrual cycles, during which nine women were administered the drugs in the order estradiol gel, placebo, estradiol gel and nine women in the order placebo, estradiol gel, placebo. The authors found that menstrual migraine attacks occurred in 30.8% of the estradiol cycles and in 96.3% of the placebo cycles. Moreover, attacks during the estradiol cycles were reported as shorter and less severe. As an acute treatment, aspirin was taken in 22 of the placebo cycles and in three of the estradiol cycles, showing a significant decline due to estradiol treatment [74].

MacGregor et al. [75] performed a crossover study consisting of 6 cycles and analyzing 35 women suffering from menstrual migraine or menstrually related migraine (numbers per group not specified). They either received a placebo gel or estradiol gel for the first three cycles, before switching this for another three cycles. The results showed a 22% (RR 0.78, 95% CI 0.62–0.99) reduction in migraine days during the estradiol cycles. These migraines were also less severe and less likely accompanied by nausea. However, after stopping the estradiol treatment, migraine frequency increased significantly for 5 days (RR 1.40, 95% CI 1.03–1.92), before decreasing to a normal state [75].

The transdermal application appears to have a lower risk of complications by maintaining near-physiological estradiol levels, compared to oral intake which is normally in higher dosages. Like in oral HRT, a progesterone component must be added in non-hysterectomized women [97]. Reported adverse events related to the transdermal supplementation were local skin reactions, increased headache, nausea, earlier bleeding, and hypertension [12].

### 4.7. Vaginal ring

The contraceptive vaginal ring is a combined hormonal contraceptive method. Due to the absorption through the vaginal epithelium, fluctuations of hormonal serum concentrations are rare [98].

Calhoun et al. [76] performed a retrospective data analysis, in which they elaborated the database of 830 women who visited a specialty headache clinic and reported high headache burden due to hormonal issues. They included 23 women in their analysis, all of which were diagnosed with migraine with aura and menstrually related migraine by a headache specialist and were prescribed extended regimen of a vaginal ring containing 0.120 mg etonogestrel/15 μg ethinyl estradiol. They found that the use of extended-cycle dosing led to a decrease in aura from 3.23 to 0.23 times per month. Moreover, menstrually related migraine had disappeared in 91.3% of the subjects. However, there are some obvious limitations in this study: its retrospective design, the sample size, and the special population of subjects, which exclusively contained women who experienced a higher burden due to their headaches than average headache burdens. Women using the vaginal ring for contraception are mostly satisfied, because of high efficacy and tolerability. The use is associated with fewer systemic side effects, such as breast tenderness and nausea, compared to combined oral contraceptives. In the above mentioned study, five women discontinued the treatment because of nausea, ring expulsion and face swelling, and abdominal pain. The risk of serious events is comparable to other combined hormonal treatments [98].

### 4.8. Subcutaneous estradiol implant

Subcutaneous estrogen implants are normally prescribed as an alternative to oral HRT in menopausal women with refractory symptoms representing a well tolerated and efficacious option [99].

One study by Magos et al. [77] examined the impact of estradiol implants on menstrual migraine. Twenty-four patients were treated with an estradiol implant for 5 years at a dose of estradiol high enough to inhibit ovulation. Of 24 patients, 23 reported an improvement in number and severity of migraine attacks. Nine patients reported a great improvement of symptoms, while eleven became completely headache free.

No adverse events were reported in this solitary study dealing with the effect of estradiol implants, highlighting the need for future studies addressing its efficacy, as well as on risks and adverse events.

### 4.9. GnRH-agonists

GnRH-agonists are used for different indications, such as prostate cancer, endometriosis, and precocious puberty. The continuous administration of larger doses results in a decline of sex hormones by inhibiting the release of gonadotropins [100].

Murray et al. performed a prospective study, including five women with severe menstrual migraine [55]. The women were first observed for 2 months and after that received leuprolide acetate – a GnRH-agonist − 3.75 mg i.m. monthly for a period of 10 months. After 5 months into the period, researchers started to administer ‘add-back’ therapy, consisting of transdermal ethinylestradiol (0.1 mg) and oral medroxyprogesterone acetate (2.5 mg), both daily, to avoid menopausal symptoms and bone loss. Women were asked to rate headache severity daily, on a scale from 0 to 3, resulting in a cumulative score for each month. They found that the mean headache score declined from 15.3 (±2.4) to 4.0 (±1.5) during the first 5 months and 3.1 (±0.7) during the ‘add-back’ therapy [55]. The study is, however, limited by its small research population, the lack of a blinded or placebo-controlled study design and application of subjective primary endpoints. Moreover, although this study did not report any adverse events, treatment with GnRH-agonists, especially long term, can result in hypoestrogenism with especially psychosocial, menopausal, and genitourinary symptoms, as well as reduced bone density [101,102].

### 4.10. Oral testosterone

Testosterone can be used in different indications. A common indication for testosterone is testosterone replacement therapy in men with hypogonadism or after bilateral orchiectomy, suffering from symptoms like erectile dysfunction, depression, loss of muscle and bone mass, etc. [103]. Testosterone is not only used in men as testosterone replacement therapy, for instance after bilateral orchiectomy [103], but also in transgender persons, which is covered more extensively later on under hormonal conditions in relation to migraine.

Glaser et al. included 27 patients in a prospective pilot study with the aim to determine the therapeutic effect of testosterone pellets, subcutaneously implanted, on the severity of migraine. Of the 27 patients, 16 were pre- and 11 postmenopausal. All patients were diagnosed with migraine and reported symptoms of androgen deficiency. They were asked to rate the severity of their migraine on a scale of 0–5 at the baseline visit and again after 3 months of testosterone therapy. After therapy, the mean severity of headaches declined from 3.63 ± 0.55 to 0.37 ± 1.08 in the combined cohort [48]. However, this study was performed without a control group, missing the ability to quantify the placebo effect. Moreover, the score used does not allow any differentiation between severity and frequency. Because of the promising results, further studies should investigate the therapeutic abilities of testosterone in placebo controlled trials, using standardized scores [48].

## 5. Future therapeutic options

While current hormonal treatments for migraine offer relief for some, a comprehensive understanding of migraine’s multifaceted nature continues to drive innovation in therapeutic strategies. In this section, we will focus particularly on the hypothalamic neuropeptide oxytocin and the pituitary-derived hormone prolactin, as these hormones have been reported to play a modulating role in migraine, contributing to its sex-dimorphism, and offer potential therapies targeting these hormones or their receptors.

### 5.1. Oxytocin

Oxytocin is of interest in hormone-related migraine, as it can suppress migraine attacks [104]. Oxytocin fibers and oxytocin receptors have been identified in several brain regions associated with migraine and nociceptive processing [105,106].

Substantial circumstantial evidence points to oxytocin playing a role in hormonal migraine pathophysiology. For instance, Amico et al. [107] found that in women chronically receiving estrogen as an oral contraceptive, oxytocin levels were enhanced. Acute ingestion of estrogen caused an increase in the level of oxytocin in plasma by 12 hours. Additionally, the perimenstrual drop in oxytocin levels in humans parallels that of estrogen and could contribute to the withdrawal trigger for menstrual attacks [43]. Similarly, the level of circulating oxytocin also increases over the course of pregnancy, while simultaneously the frequency of migraine headaches decreases [108]. Finally, breastfeeding migraine patients have higher circulating levels of oxytocin compared to migraineurs that bottle feed their baby [109] and demonstrate a slower rate of postpartum migraine recurrence [110].

Krause et al. [43] propose that estrogen acts throughout the migraine-related circuits to increase thresholds and suppress initiation of an attack, either directly or indirectly via oxytocin or other estrogen-regulated signaling molecules. Hence, an appropriately timed treatment with oxytocin agonists could form a new approach to therapy for hormone related-migraine.

Oxytocin in the systemic blood circulation, however, has difficulty crossing the blood-brain-barrier, necessitating routes other than oral or parenteral administration to reach the central nervous system [111]. An example target is the trigeminal ganglia, as in vitro experiments demonstrated that trigeminal ganglia neurons possess oxytocin receptors and are inhibited by oxytocin. Furthermore, most of these same neurons contain CGRP, the release of which is inhibited by oxytocin [112]. Decreasing the excitability of trigeminal neurons with oxytocin could lead to decreasing the probability of triggering a migraine attack.

One way of targeting trigeminal ganglia is via the nasal route, which is also more user-friendly compared to oral administration when regarding symptoms of nausea in migraine. After demonstrating that activation of central trigeminal nerve nuclei in rodents can be attenuated by intranasal oxytocin, Tzabazis et al. subsequently performed several clinical studies with intranasal oxytocin in migraine patients [104]:

A clinical pilot double-blind, placebo-controlled, single-dose study did not show a statistically significant difference in reduction of pain intensity at 2 h after administration of intranasal oxytocin compared to placebo in low-frequency migraineurs [104].

Another pilot trial was performed to test whether, in the presence of chronic migraine and thus higher levels of neuro-inflammation, analgesic efficacy of intranasal oxytocin would be increased. In this double-blind, placebo-controlled, single-dose study, patients were randomized to receive either 32 IU of intranasal oxytocin (*n* = 22) or matched placebo intranasal spray (*n* = 18). A significant difference was demonstrated 4 hours after dosing; however, there was no significant difference in the more acute measurement 2 hours after dosing. Most notable, however, 24 hours after dosing, efficacy was substantially stronger in chronic migraine patients who had not taken anti-inflammatory medication within 24 hours of oxytocin dosing, compared to participants who did take anti-inflammatory medication within 24 hours of dosing. A follow-on open-label study examining effects of 1 month of intranasal oxytocin dosing did show a reduction in pain, but a more impressive decrease in the frequency of headaches in both chronic and high frequency episodic migraineurs [104].

Lastly, a multisite double-blind, placebo-controlled, migraine prophylaxis trial was performed in Chile, Australia, and New Zealand. In total, 218 patients, including 61 high frequency episodic; 56 chronic migraineurs, were given intranasal oxytocin ‘as needed’ for 56 days, after a baseline period of 28 days. The results of this study, though not meeting its primary endpoint due to a high placebo rate in one of the countries, did show that the vast majority of patients experienced a profound increase in the number of weeks wherein no headaches were reported and so provided a clear justification for further research [104].

All in all, the above results are highly suggestive of intranasal oxytocin being effective in chronic migraine prophylaxis. More well-controlled and well-designed clinical trials with a focus on different hormonal states of patients are warranted, as well as back translational studies to understand the physiological effects of intranasal oxytocin.

### 5.2. Prolactin

Prolactin, which has a crucial role in controlling the hypothalamus-pituitary-gonadal (HPG) axis, seems to be involved in signaling mechanisms underlying migraine. A systematic review [113] investigated the role of prolactin and its receptors in headache and migraine, of which the following studies examined the serum levels of prolactin in different groups of migraine patients: A case–control study showed that prolactin levels were higher in 20 migraine without aura patients compared to 20 healthy controls on the second day of the menstrual cycle [114]; serum prolactin levels were higher in women with chronic migraine compared to women with episodic migraine [115]; no difference in serum prolactin levels in 50 female migraine patients (with and without aura) during interictal period compared to 25 age-matched healthy female controls [116]. Contrastingly, Masoud and Fakharian [117] reported that serum prolactin levels from 37 migraine patients during migraine attacks were significantly lower compared to not age-matched 37 healthy controls. The authors did not report if the healthy controls had any headache during prolactin measurement and did not compare ictal to interictal prolactin levels in migraine patients.

Regarding prolactin and migraine treatment, dopamine agonists including bromocriptine and carbidopa/levodopa have reportedly been effective in managing migraines supposedly triggered by elevated prolactin levels by inhibiting prolactin release. However, only limited evidence is yet available to support this. Hartman et al. [118] published a case-report of a 39-year-old male, with a 27-year history of chronic severe migraine, who received an incidental diagnosis of a prolactin-secreting pituitary microadenoma. Treatment of the microadenoma with bromocriptine provided complete and lasting resolution of the migraine as well, suggesting a possible etiologic relationship and prospect regarding dopamine agonist treatment in such cases of migraine influenced by prolactin.

Additionally, in a study conducted by Cavestro and colleagues [119], it was demonstrated that out of 27 patients (comprising one man and 26 women) suffering from chronic migraine, seven women exhibited elevated serum prolactin levels compared to reference values. Notably, these individuals experienced an improvement in their headache symptoms, and their migraines transitioned to an episodic pattern following treatment with the dopamine agonist cabergoline. However, there have been two cases where the administration of dopamine agonists resulted in worsening of headache [120], leaving a lingering debate in this area.

Overall, considerable evidence supports the link between elevated levels of endogenous prolactin, its receptors, and the increased incidence and severity of migraine. However, the exact underlying causative mechanisms and the difference in responses between episodic versus chronic migraine remain to be further elucidated [121]. Additional randomized and placebo-controlled clinical trials focusing on prolactin signaling are necessary to provide further insight into the role of prolactin in initiating migraine attacks and its future position in migraine treatment.

In light of this, and in order to advance toward a potential appropriately timed treatment with either existing or potential new drugs oxytocin agonists in hormone related-migraine, the effect of periovulatory administration of such migraine targeted drug treatments can be studied in a noninvasive human forehead model, developed and validated by Ibrahimi et al. [122]. The model uses a laser device as imaging technique to study local trigeminovascular effects in the forehead skin of (potential) antimigraine drugs, providing proof of target engagement in phase I clinical trials and potential guiding dose selection for phase II clinical trials. Periovulatory administration was chosen because of the ovulation coinciding with the start of the ‘estrogen withdrawal’ phase of the menstrual cycle. A secondary objective of this study is to investigate how the trigeminovascular dermal blood flow response to migraine drugs oxytocin is related to serum biomarkers, such as CGRP, serum oxytocin, and sex steroids in the study participants, further elucidating the complex interactions of several hormones in migraine.

## 6. Hormonal conditions in relation to migraine

Another challenge of the future will be dealing with patients who do not only suffer from menstrual-related migraine but also other hormonal disturbances or changes, such as patients with endometriosis, polycystic ovary syndrome (PCOS), or transgender persons receiving gender affirming hormone therapy.

### 6.1. Polycystic ovary syndrome (PCOS)

Glintborg et al. found a twofold risk for migraine in women with PCOS compared to controls [123]. However, another study consisting of 133 women with PCOS found no correlation between PCOS and the presence of migraine [124]. Some authors speculate that the higher prevalence of migraine may be due to sleep disturbances and reduced REM sleep time in PCOS [125]. What makes the comorbidity of migraine and PCOS especially challenging is that not only women suffering from PCOS have a two times increased risk of stroke and thrombosis [123] but also the first-line treatment for PCOS, combined oral contraceptives, is associated with a worsening in migraine and a higher risk of stroke [126]. Therefore, we encourage the use of non-hormonal treatments for women with PCOS and migraine, such as lifestyle changes and metformin, which have shown to be effective [127].

### 6.2. Endometriosis

Endometriosis is significantly associated with an increased risk of migraine headache, according to a recent meta-analysis, which included 287.174 women (OR 1.56 95% CI 1.21–1.90) [128]. Some biochemicals such as prostaglandins, nitrite oxide, and estrogen are involved in the pathophysiology of both diseases [128]. Estrogen seems to play a central role in the genetic link underlying the comorbidity of endometriosis and migraine. Women who share overlapping estrogen receptor polymorphisms may find greater relief from their symptoms through continuous progestin-based estrogen suppression. Several studies show that treatment with progestin-only options has a beneficial effect on both migraine and endometriosis, as reviewed by van der Vaart et al. [129], whereas combined oral contraceptives often worsen migraine symptoms [10]. Identifying the co-occurrence of migraine and endometriosis can therefore optimize hormonal treatment and we recommend treating migraine in women with endometriosis with oral progestins or levonorgestrel intrauterine system as firstline treatment and GnRH-agonists after treatment failure [130]. Combined oral contraceptives or danazol are not recommended [131].

### 6.3. Transgender persons receiving gender affirming hormone therapy

In transgender men (i.e. female at birth who identifies as a male), testosterone is used as a treatment to induce masculinizing changes, called gender affirming hormone therapy [132]. Finasteride, a 5α reductase inhibitor, is mostly used in benign prostatic hyperplasia by lowering serum levels of dihydrotestosterone [133].

In addition, it can be used in transgender women (i.e. male at birth who identifies as a female) for gender affirming hormone therapy [132]. In an anecdotal report published by Check and Cohen, the administration of 5 mg finasteride, a 5α reductase inhibitor, in a woman with chronic migraine, led to full disappearance of symptoms. After stopping the therapy due to dry eyes, the migraine returned [134]. No evidence was found regarding the risk of stroke or thromboembolic events.

Another case report includes two transgender persons receiving gender-affirming hormone therapy, or more specifically, masculinizing hormone therapy with testosterone. They were both diagnosed with chronic migraine without aura. Headache severity was reported by using the Migraine Disability Assessment Score (MIDAS) and frequency by using monthly migraine days. After starting a therapy with injection of 200 mg/mL testosterone enanthate every 14 days, the first patient reported an improvement concerning the MIDAS from 46 to 5. The second patient was treated with 50 mg testosterone enanthate, later increased to 100 mg. Through this therapy, the patient reported a decrease in MIDAS from 17 to 8 and experienced a total cessation of menstrual bleeding [135].

A cross-sectional study on the effect of gender transition on primary headaches found that 50% of transgender males with migraine using gender affirming hormone therapy reported a decrease in migraine frequency, while 43.8% did not experience any change [136].

The use of testosterone as gender affirming hormone therapy in transgender men may be associated with a higher cardiometabolic risk. Two prospective studies found that after 1 year of use, subjects had higher levels of total cholesterol and LDL-cholesterol as well as lower levels of HDL-cholesterol [137,138]. These changes were confirmed by a meta analysis including 1500 transgender men, which also showed that a longer duration of use (>2 years) is associated with more undesirable effects on the lipid profile [139]. Additionally, some data suggest that gender affirming hormone therapy with testosterone could increase blood pressure; however, results are mixed and further studies are needed [132]. However, according to current evidence, the risk of stroke does not seem to be elevated by testosterone given as gender affirming hormone therapy, compared to cisgender men and women [140].

### 6.4. Breast cancer

Breast cancer can be divided into four molecular subtypes, i.e. luminal A, luminal B, human epidermal growth factor receptor 2 (HER2), and basal-like breast cancer. About 60–70% belong to the luminal-like breast cancers, which are highly influenced by hormonal factors, expressing an estrogen receptor [141].

Migraine and breast cancer both have been shown to exhibit strong relations to the endocrine system. It has been a matter of discussion whether there is an association between migraine and breast cancer – a conflict further fueled by the fact that data on this topic are very heterogeneous. Several studies [142–144] have supported the hypothesis of migraine having beneficial effects by reducing the risk of breast cancer.

However, a recent prospective study [145] found quite the opposite. Researchers detected a highly elevated prevalence of migraine in women with breast cancer, especially in the luminal-like subtypes. While the prevalence in the general population is about 17%, in this study 56% of participants were diagnosed with migraine, 76% of those with migraine without aura. Moreover, more than 50% of study participants suffering from migraine reported it to be menstrually related. Interestingly, migraine patients had a higher risk of getting diagnosed at stage II or III breast cancer than at stage I. Further, while 365 of 440 women were treated with hormonal therapy, mostly aromatase inhibitors (47.4%) and tamoxifen (35.9%), researchers detected no influence of the hormonal therapy on the course of migraine.

The discrepancies in data concerning the association between migraine and breast cancer might be explained by methodological differences between the studies. Only few studies used the ICHD classification for migraine diagnosis, opening the door to misdiagnosis and making a comparison of the outcomes of the different studies impossible. Another important aspect is the fluctuating character of migraine. Theoretically, it is possible that a woman may have suffered from migraine for years but is headache-free at the time of the breast cancer diagnosis, and therefore, it goes unnoticed as she reports not to have migraine. To date, there is no clear answer to how migraine and breast cancer influence each other and what the underlying mechanisms to that might be [146].

## 7. Conclusion

The abovementioned comorbidities illustrate the need for increased awareness that there is no one-size-fits-all approach in hormone-related migraine. While we did not conduct a systematic review and we do not claim that the current review includes all studies in the field, we observed that data are limited and suggestions for treatment are sometimes based on weak evidence and studies with limited power. Nonetheless, women with menstrual migraine are recommended to not use the standard regimen of the combined oral contraceptive pill, but rather an alternative regimen or use hormonal treatments based on progestins, like the progestin-only pill or the levonorgestrel intrauterine system. These recommendations should be underlined by further studies based on standardized parameters. Moreover, great effort should be put into the research on future therapeutic options, in order to enlarge the number of options. As societal diversification advances and the number of patients suffering from multiple comorbidities keeps growing, the migraine patient population will become increasingly complex. Therefore, a larger variety of safe, efficacious, and personalized treatment options are becoming increasingly important.

## 8. Expert opinion

Migraine is a complex and debilitating neurovascular disorder, in which finding the right treatment pattern is not only challenging for clinicians but also crucial for the patient’s quality of life. In the last years, much attention of research has been given to the CGRP(−receptor) monoclonal antibodies and gepants, which undoubtedly enhance the quality of life for many patients. However, the treatment of menstrual migraine still poses specific challenges, being a more resistant subtype that is less responsive to prophylactic pharmacological treatments [147]. Results of a recent study on the effect of CGRP(−receptor) monoclonal antibodies in 40 patients with menstrual-related migraine and three or more previous treatment failures further confirmed this [148]. Besides a significant reduction of median menstrual migraine frequency, pain intensity, and attack duration, they showed that menstrual migraine episodes had a slightly less treatment response compared to the non-menstrual migraine episodes (72.4% reduction vs. 60% reduction) [148]. Notably, post-hoc analyses of a real-world study showed no differences in treatment responses between perimenstrual and nonperimenstrual migraine days in menstruating women using erenumab or fremanezumab, although the treatment effect was greater in women compared to men [149].

The difficulty of treatment in these patients, due to different treatment responses is shown in our review, and the data presented draws a difficult picture. As there are only very limited data available, and quality of evidence is weak due to missing out on randomized controlled trials, studies with sufficient power and standardized parameters to ensure standardized assessment of the treatment effects, recommendations can only be given with reservation.

This proves that the demand for future studies and future therapeutic options for menstrual migraine is high. Upcoming studies should evaluate efficacy and safety of treatment options (e.g. oxytocin) by using comparable and objective end points like monthly migraine days and specific pain scores [21]. Moreover, more randomized controlled trials consisting of a proper design and cohort studies with adequate power are needed. As our review has presented studies with conflicting results, it once again pointed out the importance of individual therapy considerations and personalized medicine. As we have seen in treatment forms like CGRP(−receptor) or especially gepants, pharmacokinetic and pharmacodynamic processes play a pivotal role in treatment responses, efficiency, and side-effects in individual patients. These processes are influenced by patient characteristics like age, sex, gender, and ethnic background – underlying the importance of adhering to an intersectional framework when developing individualized treatment recommendations [150,151].

While the available treatments very often show unsatisfying results, oxytocin and prolactin show potential as therapeutic options in the future. Being currently researched, smaller studies have already indicated that they could have beneficial effects on migraine and, therefore, could represent new targets to tackle the migraine burden [152]. Exploring future treatment options for migraine is also imperative due to the need to mitigate potential long-term cardiovascular risks associated with CGRP(−receptor) targeted antibodies [153], and the combined hormonal treatments available [87].

However, this should not detain researchers from examining the effect of already existing therapies and off-label use of hormonal therapies on the course of migraine. As an example, aromatase inhibitors – which suppress estrogen serum levels and are currently approved as a treatment of (hormone-receptor positive) breast cancer in premenopausal (in addition to a GnrRH analog) and postmenopausal women and in men and gynecomastia in men [154] – might theoretically be an attractive treatment option in men with chronic migraine. Indeed, these individuals exhibit higher serum estradiol levels and a lower estradiol/free testosterone ratio [37], suggesting a hormonal influence of estrogen in particular. In addition, although unsupported by actual published data, the aforementioned pilot study of Glaser et al. [30] mentions that many premenopausal patients were found to have a larger headache relief with the addition of the aromatase inhibitor anastrozole delivered in combination with testosterone. Therefore, a further exploration of the use of aromatase inhibitors, especially in those who suffer from comorbidities (e.g. breast cancer), could potentially serve as a valuable agent in the field. Studying their clinical effects could, therefore, provide additional insights into the modulating role of estrogen levels and their impact on migraine frequency and severity in both male and female migraine patients.

Another priority of the future will be to conduct rigorous research on the pathophysiology of menstrual migraine to understand the interaction between different sex hormones while disentangling their individual contribution. A translational study found reduced trigeminovascular cyclicity in women with menstrual migraine, with the trigeminovascular activity being reduced during the first days of the cycle, compared to healthy controls [36]. This is indeed notable, as the higher CGRP concentrations in the blood and tear fluid during migraine attacks would lead to the assumption of higher activity during these migraine likely days [34]. These results are exemplary for mechanisms not being fully understood, while understanding the pathophysiological backgrounds would enable researchers to detect more specific targets.

In summary, migraine, a complex condition, is frequently accompanied by comorbidities, emphasizing the imperative for personalized medicine. This approach entails a deep comprehension of a patient’s unique hormonal sensitivities, medical history and comorbidities, as well as risk profile. Coupled with rigorous research and the advancement of novel hormonal targets, personalized medicine holds the key to pave the way to a migraine-less future.

## Article highlights

- Migraine is a common neurovascular disorder characterized by recurrent moderate-to-severe headache attacks. Migraine has an estimated prevalence of 15% worldwide.
- Recent advances in therapies involving CGRP blockade are not as effective in more resistant subtypes of migraine, such as menstrual migraine.
- Future research should, therefore, consider the pathophysiological mechanisms involving complex interactions between sex hormones and CGRP in migraine while considering patient-specific characteristics.
- Well-designed clinical trials are warranted to evaluate the efficacy and safety of promising future hormonal treatments in migraine, such as oxytocin and prolactin.
- The current gap in literature underlines the importance of a personalized and intersectional approach based on specific characteristics such as age, sex, gender, ethnicity, (hormonal) medical history, and current hormonal profile.

## Appendix

(migraine/mj/exp OR (migrain*):ti,kw) **AND** (‘menopause and climacterium’/exp OR (menopau* OR climacteri* OR premenopau* OR perimenopau* OR postmenopau* OR perimenstrual* OR peri-menstrual*):ab,ti,kw) **AND** (therapy/exp OR ‘hormonal therapy’/exp OR ‘oral contraceptive agent’/exp OR ‘migraine’/exp/‘drug therapy’ OR (therap* OR treatment* OR management* OR ((hormon*) NEAR/3 (contracept* OR substitut* OR exogen* OR manipulat* OR manag* OR replac*)) OR ((estrogen* OR oestrogen*) NEAR/3 (therap* OR treatm*)) OR ((oral* OR pill OR drug*) NEAR/3 (contracept* OR anticoncept*)) OR HRT):ab,ti,kw OR (estrogen* OR oestrogen*):ti) NOT ([Conference Abstract]/lim OR [Conference Review]/lim) NOT ([animals]/lim NOT [humans]/lim) AND [ENGLISH]/lim

## Medline

(exp *Migraine Disorders/OR (migrain*).ti,kf.) **AND** (exp Menopause/OR (menopau* OR climacteri* OR premenopau* OR perimenopau* OR postmenopau* OR perimenstrual* OR peri-menstrual*).ab,ti,kf.) **AND** (therapy.fs. OR exp Hormone Replacement Therapy/OR exp Contraceptives, Oral/OR (therap* OR treatment* OR management* OR ((hormon*) ADJ3 (contracept* OR substitut* OR exogen* OR manipulat* OR manag* OR replac*)) OR ((estrogen* OR oestrogen*) ADJ3 (therap* OR treatm*)) OR ((oral* OR pill OR drug*) ADJ3 (contracept* OR anticoncept*)) OR HRT).ab,ti,kf. OR (estrogen* OR oestrogen*).ti.) NOT (news OR congres* OR abstract* OR book* OR chapter* OR dissertation abstract*).pt. NOT (exp Animals/NOT Humans/) AND english.la.

## Cochrane

((migrain*):ti) **AND** ((menopau* OR climacteri* OR premenopau* OR perimenopau* OR postmenopau* OR perimenstrual* OR peri-menstrual*):ab,ti) **AND** ((therap* OR treatment* OR management* OR ((hormon*) NEAR/3 (contracept* OR substitut* OR exogen* OR manipulat* OR manag* OR replac*)) OR ((estrogen* OR oestrogen*) NEAR/3 (therap* OR treatm*)) OR ((oral* OR pill OR drug*) NEAR/3 (contracept* OR anticoncept*)) OR HRT):ab,ti OR (estrogen* OR oestrogen*):ti) NOT “conference abstract”:kw

## Web of Science

TI=(migrain*) **AND** TS=(menopau* OR climacteri* OR premenopau* OR perimenopau* OR postmenopau* OR perimenstrual* OR peri-menstrual*) **AND** (TS=(therap* OR treatment* OR management* OR ((hormon*) NEAR/2 (contracept* OR substitut* OR exogen* OR manipulat* OR manag* OR replac*)) OR ((estrogen* OR oestrogen*) NEAR/2 (therap* OR treatm*)) OR ((oral* OR pill OR drug*) NEAR/2 (contracept* OR anticoncept*)) OR HRT) OR TI=(estrogen* OR oestrogen*)) NOT TS= ((animal* OR rat OR rats OR mouse OR mice OR murine OR dog OR dogs OR canine OR cat OR cats OR feline OR rabbit OR cow OR cows OR bovine OR rodent* OR sheep OR ovine OR pig OR swine OR porcine OR veterinar* OR chick* OR zebrafish* OR baboon* OR nonhuman* OR primate* OR cattle* OR goose OR geese OR duck OR macaque* OR avian* OR bird* OR fish*) NOT (human* OR patient* OR women OR woman OR men OR man)) NOT DT=(Meeting Abstract OR Meeting Summary)

## Google Scholar

migraine menopause|premenopause|perimenopause|postmenopause therapy|treatment|“hormone contraceptive|substitution|replacement”|“oral contraceptive|anticonception” -animal -mouse -mice -rat

migraine menopause|premenopause|perimenopause|postmenopause therapy|treatment|‘hormone contraceptive|substitution|replacement’|‘oral contraceptive|anticonception’ -animal -mouse -mice -rat
